# Directing and Orienting ICT Healthcare Solutions to Address the Needs of the Aging Population

**DOI:** 10.3390/healthcare9020147

**Published:** 2021-02-02

**Authors:** Nada Fares, R. Simon Sherratt, Imad H. Elhajj

**Affiliations:** 1 Department of Biomedical Engineering, School of Biological Sciences, University of Reading, Berkshire RG6 6AY, UK; n.fares@pgr.reading.ac.uk; 2 Department of Electrical and Computer Engineering, American University of Beirut, Beirut 1107 2020, Lebanon; imad.elhajj@aub.edu.lb

**Keywords:** assistive/home monitoring technologies, older people, caregivers, activities of daily life, assistive living, fall detection, fall prediction, fall prevention, chronic diseases

## Abstract

Background: With an aging population, it is essential to maintain good health and autonomy for as long as possible. Instead of hospitalisation or institutionalisation, older people with chronic conditions can be assisted in their own home with numerous “smart” devices that support them in their activities of daily living, manage their medical conditions, and prevent fall incidents. Information and Communication Technology (ICT) solutions facilitate the monitoring and management of older people’s health to improve quality of life and physical activity with a decline in caregivers’ burden. Method: The aim of this paper was to conduct a systematic literature review to analyse the state of the art of ICT solutions for older people with chronic conditions, and the impact of these solutions on their quality of life from a biomedical perspective. Results: By analysing the literature on the available ICT proposals, it is shown that different approaches have been deployed by noticing that the more cross-interventions are merged then the better the results are, but there is still no evidence of the effects of ICT solutions on older people’s health outcomes. Furthermore, there are still unresolved ethical and legal issues. Conclusion: While there has been much research and development in healthcare ICT solutions for the aging population, ICT solutions still need significant development in order to be user-oriented, affordable, and to manage chronic conditions in the aging wider population.

## 1. Introduction

Aging is an instinctive, disruptive, and wide-ranging process for every individual. Aging is a process distinguished by different morphological, pathophysiological, psychological, social, and environmental aspects. All these aspects of aging are inspected in “gerontology”. Social and cultural gerontology has symbolised the assumption of aging as a biomedical process while focusing on the contextual factors that mould the practices and patterns of aging [1]. The age of the person is assessed in each aspect by their functional abilities. Therefore, a person’s health status is the consequence of the accumulative effects of aging in addition to the obtained disease. This aging situation leads the person to a state of “unstable incapacity” for “normal aging” [2]. The aging process is specified in older people by a decreased reaction to the environment as well as to definite pathologies accompanied by an obvious decrease in individualism and incorporation in activities of daily living (ADL). The aging process affects various systems in the human body, such as the nervous system, sensory system, cardiovascular system, metabolic system, respiratory system, and musculoskeletal system. The observed effects of the aging process are more visible in the musculoskeletal system in older people in terms of mobility and locomotion [3]. Each year, 37.3 million falls are encountered among older people aged 65 and above. This makes older people more vulnerable to fatal falls that require medical care [4]. Although falls are unavoidable in nature, it is possible to detect, manage, and even prevent them from occurring by means of proper administration of human and technical interventions. This dual embrace between gerontology and technology is regarded as “gerontechnology”. The aftereffect of this routine is to be recognised in terms of an increase in independence and an improvement in the quality of life of older people [5]. This indicates the importance of science and the design and use of technology in the development of ICT solutions that focus on user involvement, viewing the older person as an agent that creates and develops meaning for later life as they interact with technology [1]. However, the health of older people is known to be heterogeneous in nature. Heterogeneity in the health of older people is such that differences among people of the same age may be greater than those inferred from chronological age differences, based on the number of years a person has been alive. There are different factors affecting the health outcomes of older people. These factors can be biomedical (e.g., genetic), socioeconomic, or behavioural, such as exercise, nutrition, social engagement and support, stress levels, and geographic location. In this research, we focus on the biomedical perspective of aging excluding other factors of heterogeneity affecting the health of older people. Therefore, the user of ICT solutions is limited to older people with chronic diseases. The involvement of this group of older people in the creation of ICT healthcare solutions allows for the development of solutions which are personalised. The challenge here for researchers is to find the tools and channels to innovate approaches and technologies focusing on the user-centred needs of older users with chronic diseases [1].

Several smart ICT solutions have already been commercially distributed. Such solutions include all electronic products that deal with information in a digital form as storing, processing, transmitting, converting, duplicating, or retrieving electronic information. Examples of ICT solutions are communication devices or applications as radio, television, computer network, satellite system, the internet, smartphones, and personal digital assistants (PDA). ICT systems in the healthcare sector for older people monitor and manage various aspects of the aging process in older adults. Common aspects of the aging process from a biomedical perspective include hearing loss, musculoskeletal deterioration causing falls and near-falls, chronic obstructive pulmonary disease, heart disease, diabetes, and dementia. Older adults are more likely to experience more than one condition at the same time.

The monitoring and management process of the different aspects of aging is carried out through using different kinds of sensors to capture motion data, biological data, and environmental data for more in-depth data analysis and processing. Falls and the resulting injuries encountered in the aging population manifest their occurrence due to aging, chronic disease-related medical conditions, and the older people’s interactions with their social and physical environments [6]. Falling risk escalates when multiple risk factors are interconnected [7]. This confirms the need for healthcare-monitoring ICT solutions for older people that manage their health and consequently prevent health decline and falls and detect near-fall events.

Other aspects of the aging process such as natural cognitive and psychical decline are also monitored and managed using ICT solutions addressing different chronic diseases. The cognitive and physical decline in turn impacts the functional abilities and increase the risk of falls and near-falls in the aging population [8]. This imposes new challenges on the socioeconomical level of the increased demographic change in the population. These new challenges pinpoint the need for appropriate management of older people’s health to reduce the impact of unavoidable aging and its corresponding fallouts.

Aging of the population and the onset of chronic diseases are interconnected. The development of ICT healthcare solutions impacts the quality of life (QoL) of older people through serving independent living at home as well as eliminating health and social expenses in the aging population. Thus, the innovation of this paper lies in the analysis of health-related ICT solutions with the aim of identifying their effectiveness in encouraging active and independent living of older people at home, satisfying the needs of the older people and their caregivers, improving their wellbeing and QoL, and making it affordable [9].

**Figure 1:**
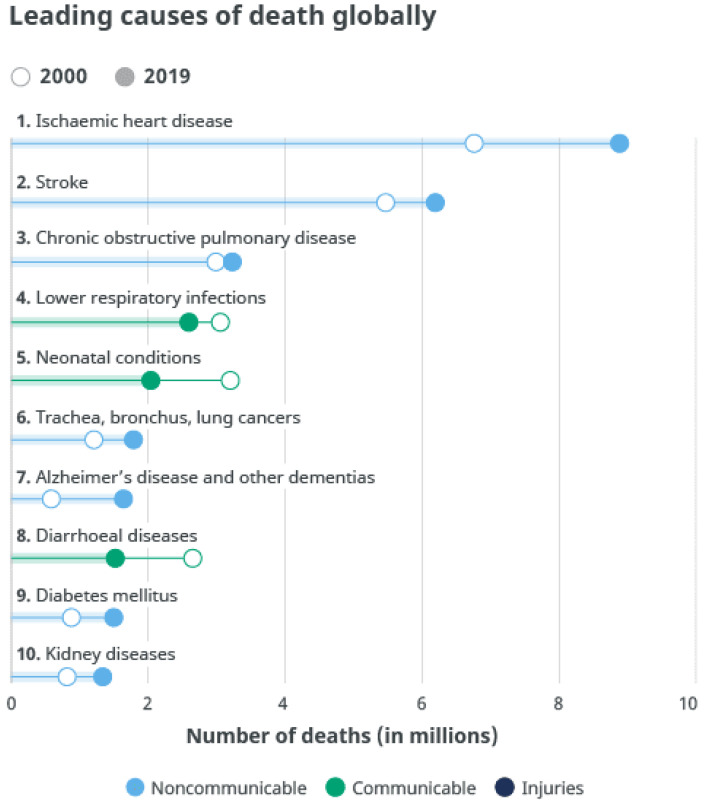
Chronic diseases causing death—top 10 global causes of death, 2016 [16].

There are many proposed health-related ICT solutions for the aging population. Such solutions are used in the framework of monitoring older people’s activities [10,11,12] or monitoring and managing their health [13,14,15]. These solutions make it possible for the older person to use wearable sensors, environmental sensors, and devices that are devoted to monitoring and/or managing their health conditions in relation to the top ten chronic diseases causing death globally, as identified by the World Health Organization (WHO) [16], as presented in Figure 1. Multiple factors contribute to mortality in older people. To predict mortality in older people, different characteristics as the obtained chronic disease, functional abilities, and personal characteristics of the older person need to be identified and analysed. This review is limited to a biomedical perspective and does not focus on environmental and socioeconomical factors such as pollution, chemical exposure, social isolation, sex, and unhealthy habits causing mortality in the older population. Various technologies are adopted in the assembly of ICT solutions with different characteristics as user friendly configuration, smart home integrated management, and social community. To support the older person’s needs and independence of the aging population, these technologies consist of sensor or device-controlled functions, communication and connection methods, a cloud, and analytics integrated into the living environment of the older person [17]. Using this methodology, better coordination between caregivers, emergency response teams, and intervention can be achieved. This approach serves to reduce costs and improve QoL for older people having chronic medical conditions.

In this review, it is concluded that older people do accept smart home technologies if they permit their independent living in their own environment. Older people’s perceptions of ICT solutions at home have emerged from their belief that such technologies could invest in various aspects of independent living. Such aspects include providing support in emergency situations, assistance with medical conditions as hearing and visual impairment, detection and prevention of falls and near-falls, monitoring of physiological specifications as blood pressure and glucose level, assurance of older persons’ safety and the security of their living environment, and finally providing a reminder system for appointments and medication and contradictions, if any [18].

This review considers the current state of the art of the available ICT solutions for healthcare monitoring in older people. Different categories of ICT solutions with different approaches for healthcare monitoring of older people are presented to reflect the available gaps and hypothesise requirements to be present in future ICT solutions for older people’s healthcare management. Section 2 presents the research methodology. Section 3 discusses different ICT categories, development processes, and architectures applied in healthcare. Section 4 provides a presentation of the state of the art in ICT healthcare solutions for assistive living in the aging population and presents the results of the review by evaluating different ICT solutions’ readiness, acceptance by users, and effectivity in improving the older person’s quality of living. Section 5 discusses the implications of the review and how ICT technologies can be improved to meet the needs of the aging population. Section 6 concludes the present study and offers future research directions.

## 2. Materials and Methods

A systematised literature review was carried out to recognise and assort the available ICT technologies. A PRISMA (Preferred Reporting Items for Systematic reviews and Meta-Analysis) [19] Protocol-based scoping review was conducted using a full multiple reviewer [20]. Preliminary research and idea validation were first conducted to identify relevant articles, ensure the validity of the proposed idea, avoid duplication of previously addressed questions, and ensure the availability of enough articles for conducting the systematic review. This was accomplished by performing a simple search in various resources such as PubMed and Google. The sources adopted in this review are PubMed, ScienceDirect, Springer, Google Scholar, and IEEE Xplore. Journal papers and conference proceedings were searched using the keywords “Assistive/home monitoring technologies” OR “ICT” OR “smart systems” AND “older people”, “older adults”, “caregivers”, AND each of the category-based search terms below:

- Activities of daily life, assistive living, independent living, quality of life;
- Smart homes, health monitoring, healthcare solutions;
- Disability, mobility, autonomy. Fall detection, fall risk assessment, fall prediction, fall prevention;
- Chronic diseases, dementia, Alzheimer’s disease, cognitive impairment, heart disease, diabetes, COPD.

**Figure 2:**
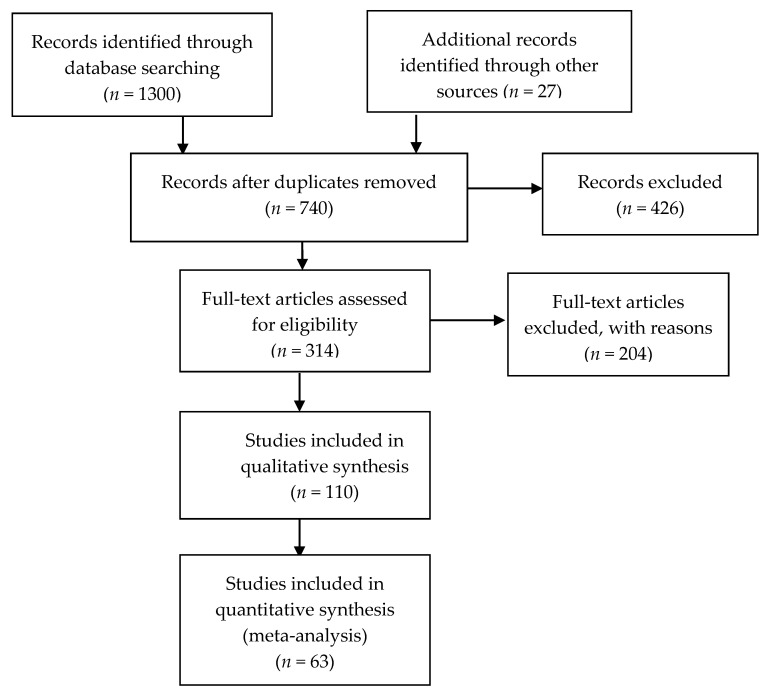
PRISMA flowchart for screening literature.

A total of 1327 papers were screened (based on titles and abstracts) to determine their relevance to the research topic. Then, 426 papers were excluded due to their irrelevance, duplication, or unavailable full texts. One hundred and three papers were included and evaluated due to their focus on relevant healthcare issues, consideration of the global needs of older adults, and consistency with the adopted review methods. Finally, only 63 papers were selected due to their inclusion of information that answers our research question. The chosen papers cover studies with direct relation to ICT solutions for assistive living of older people in their own environment, excluding topics covering ICT solutions using invasive technologies, systems based on gaming technologies as Wii and Xbox, and clinical solutions. Figure 2 presents a PRISMA flowchart for screening the literature in this research review.

## 3. ICT Solutions

In the field of healthcare advancement, ICT solutions may potentially play an important role in enhancing the quality of life of the aging population and allow their independent living. Integrated ICT solutions assist in the healthy and safe aging of older people and minimise health and social expenses. They are greatly sustained in developed countries aiming to improve the quality of life (QoL), ensure the sustainability of care of its aging population, and pursue the demographic crisis through applying ICT solutions for home care in the context of old age-related chronic diseases [19].

### 3.1. ICT Health Monitoring Systems

#### 3.1.1. Healthcare Monitoring and Management Architecture

**Figure 3:**
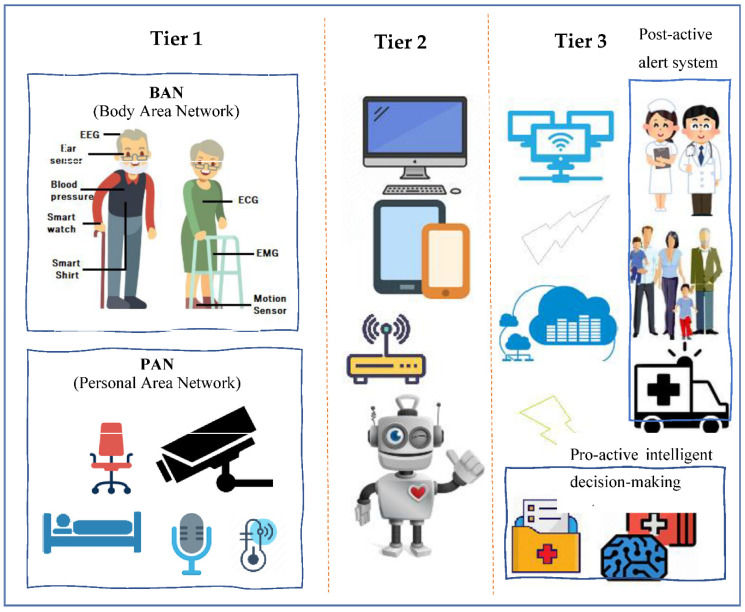
Common healthcare monitoring systems architecture.

Various healthcare ICT solutions have been investigated in this review. It was recognised that these solutions shared a common architecture and a similar set of properties. The common ICT solution architecture involved three tiers that collaborate to collect and analyse the sensory data of an older person. The tiers include consequently a set of sensors, a gateway, and a remote monitoring/management system. The different components of the tiers and how they collaborate are presented in Figure 3. In this section, the progression of the common healthcare monitoring and management architecture is portrayed, and the deployed ICT technologies are described.

##### Tier 1—Sensors

This tier is formed from various sensors that collect physiological, body motion, or environmental data. The sensors are devoted to the collection of signals attached to the Body Area Network (BAN) of the user and/or are arranged as a Personal Area Network (PAN) or ambient sensors in the home of the user [21]. Sensors in a BAN are attached directly to the body of the older user, to his/her clothes, or implanted under the skin. BAN sensors constantly collect physiological signals from the older user. Such signals correspond to the temperature, heartbeat, physical activity, blood pressure, electroencephalography (EEG), electromyography (EMG), and electrocardiography (ECG) [22].

A PAN is developed of ambient sensors positioned on devices used by the older user. Such sensors provide rich contextual information about the user’s activities and his/her living environment. Sound sensors, video cameras, RFID readers, pressure sensors, luminosity sensors, proximity sensors, pressure sensor, temperature sensors, humidity sensors, and location tracking sensors are examples of PAN sensors [22]. The extracted data from the sensors are in turn broadcasted to a local device using different methods.

##### Tier 2—Gateway

A gateway can be a customised device or a smartphone that is in the older user’s living environment [22]. A gateway usually has the power of collecting data from sensors and dispatching them to a remote server placed either at the hospital or on a cloud to be analysed. However, in later approaches, with the evolution of fog computing, analysis of the collected data is performed locally in the living environment of the older user for accelerated decision-making and better implementation of bandwidth where only partial data updates are transmitted to the cloud for further analysis or for historical recording.

##### Tier 3—Remote Monitoring, Feedback, and Decision-Making System

Monitoring, feedback, and decision-making are usually performed on a remote system (server) considered the core of the ICT system to which various data (physiological and environmental data) from sensors are transmitted [22]. Such a system has the following functionalities: (i) Operate specific algorithms on the collected data for further interpretation and analysis; (ii) Issue alerts in case of emergencies; (iii) Record every user’s data to be stored in a database for future analysis as the prediction of undiagnosed diseases by applying data mining techniques; and iv) Offer a Graphical User Interface (GUI) to enable users (older people, caregivers, and clinicians) to monitor real-time health status.

#### 3.1.2. Classification of ICT Healthcare Monitoring and Management Solutions

ICT healthcare monitoring and management systems are categorised into wearable based systems (WS), non-wearable-based systems (NWS), and fusion or hybrid-based systems (FS) [23]. The quantity and type of deployed sensors depends mainly on the context of the performed application, i.e., user’s activity monitoring, older user’s health monitoring and management, or both. Various types of sensors are assembled in wearable, non-wearable, and fusion systems.

(1) Wearable System Sensors: Wearable system sensors consist of a collection of inertial and non-invasive sensors placed on the body of the older user. Wearable-based systems extensively use inertial sensors as accelerometers, gyroscope, inclinometer, barometers, and magnetometers to detect unexpected escalation in human gait, assess balance, and monitor displacement [24]. Inertial sensors are basically utilised for the identification of different causes of falls in the aging population, i.e., falls from sleeping, falls from sitting, falls caused by walking or standing, and falls caused by using supportive tools as a ladder, walker, or stairs [25]. For more precise monitoring in the context of fall prevention, more motion sensors are being placed on different body parts with the aim of measuring a wider range of characteristics of the human gait [26]. Such sensors are extensometers, force sensors, and goniometers. These sensors provide input linked to human locomotion such as extracting different gait characteristics and detecting abnormal gait behaviour. On the other hand, intrinsic factors associated with the pathophysiological history of the older user as muscle fragility, diabetes, heart diseases, and hypertension can cause a change in the older user’s behaviour and may lead to a fall or near-fall [27]. Non-invasive healthcare sensors are deployed to collect healthcare data from the older user. Such healthcare data can be blood glucose, blood pressure, cortisol [28], agitation detection [29], and cardiac activity collected and measured using intrinsic sensors as infrared sensors, optical sensors, and oscillometers [30].
(2) Non-Wearable System Sensors: Non-wearable system sensors consist of sensors deployed in the living environment of the older user. Such sensors serve privacy issues when obstructiveness is rejected by the older user. These systems have the aim of tracking user’s activities of daily living (ADL) and detecting unexpected changes in motion. However, the functionality of such solutions is limited to the sensor’s coverage range [31]. Sensors deployed in this category of systems are pressure, motion passive infrared (PIR), vibration, acoustic, and infrared sensors. These sensors are more appropriate for older people living at home and healthcare accommodations where falls are more likely to occur. According to the US Dept. of Health and Human Services [32], cognitive and physical decline in older people is inspected through the detection of a change in the behaviour of the older user that is associated with gait deficits and subsequent falls. Non-wearable systems are correlated with identifying abnormalities, tracking any change in gait parameters, and detecting emergencies [23].
(3) Fusion System Sensors: Various types of wearable and non-wearable sensors are interconnected in a hybrid sensing system to provide a multichannel source of data to be analysed using single or multiple algorithms. Koshmak et al. [33] explained that the fusion of sensors can reasonably provide a significant improvement in fall-related systems in terms of reliability and specificity since the multisensory fusion approach meets the needs of older people living independently. The aim of deploying a fusion system for sensing is the flexibility of adjusting the system to a wider context such as a wider sensor network, implementation of fall detection and prevention strategies, designing a user-oriented monitoring and management process, and scalability. Currently, fusion systems are mainly utilised in human gait assessment labs with efforts to export such sensing technologies into the living environment of the older user, i.e., allowing ambient assisted living (AAL) for the aging population. A new ICT trend involves intelligent objects which are installed in the living environment of older people to support their independent living and the monitoring process. With all the advantages of fusion systems in the monitoring and management of older people’s health, they still face some challenges such as permitting their integration into smart homes, performing real-time analysis, improving their computational power, and reducing their cost [23].

Smartphone sensing technologies are considered as either wearable or non-wearable technologies that are competent in sensing, processing, and communicating user’s data while living in their own environment. These smartphone systems are either used as stand-alone sensing systems or are connected to other wearable or non-wearable sensors for more detailed monitoring and management of the older user’s health as well as the detection and prevention of falls. Savenstedt et al. [21] declared that smartphone-based systems are still not yet deployed as stand-alone systems in a non-wearable context.

#### 3.1.3. Data Processing Techniques in ICT Solutions

**Figure 4:**
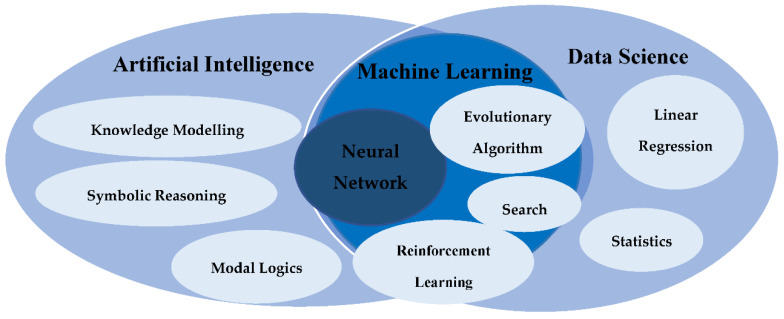
Data processing in ICT.

Data processing is a mechanism that includes the redeeming, remodelling, or categorising of data. Data processing is mainly dependent on the volume of data, the complexity of the performed processing operations, the inbuilt technology of the operating system, and time restrictions. Examples of data processing techniques are data indexing, data mining, image processing, and video transcoding. Such techniques rely on the data parameters collected from different data sources. In different ICT solutions, i.e., wearable, non-wearable, or fusion ICT solutions, data parameters are extracted mainly from sensors. The data to be processed can be either human-generated as images, audio, or video files or machine-generated as log files generated by operating systems and network management log files, although processing is carried out by the machine. The data processed are either processed in patches when similar data are grouped and processed together or in real-time where data are processed immediately. Figure 4 shows how artificial intelligence, machine learning, and data science are interconnected to provide data processing techniques that serve the accurateness of the processed data.

Artificial intelligence and data science are a broad domain of applications, systems, and more that target the replication of human intelligence through machines. To help computers learn automatically, artificial intelligence binds large amounts of data through iterative processing and intelligent algorithms. Artificial intelligence uses logic and decision trees. Data science deals with both structured and unstructured data and operates by sourcing, cleaning, and processing data to derive meaning out of them for analytical purposes.

Analytical methods (ANS) and machine learning methods (MLM) are methods used in data processing [23]. Analytical methods are derived from classic techniques which use statistical models to obtain clarification from data for prediction. Linear regression, time series, and transformations are examples of the analytical methods performed. ICT solutions based on ambient sensing systems use event sensing techniques. This sensing technique is carried out using vibrational data that are useful for the monitoring, tracking, and localisation of the user of the system. ICT solutions with cameras perform image processing techniques. Different types of image processing methods are performed based on the requirements of the data to be collected. For example, to recognise the lying or standing posture of a user, spatiotemporal features of the user are extracted while in scene, i.e., the weight, skin colour, ratio of silhouette height, width, and orientation of main body axis.

On the other hand, machine learning methods depend on complex algorithms to achieve a closer interpretation of the collected data with the aim of predicting output decisions as in neural networks. Neural networks allow the operating system to learn from observational data. Neural networks are considered of the best machine learning methods since they address many challenges in image recognition, speech recognition, and natural language processing. As for camera-based fall detection ICT systems to achieve insight from the data for fall detection and even prediction, different methods that can be used are support vector machine (SVM) techniques as presented in Sakr et al. [34], Gaussian distribution of clustered knowledge, naïve Bayes, multilayer perception, and decision trees [29].

### 3.2. Different Categories of ICT Solutions

**Figure 5:**
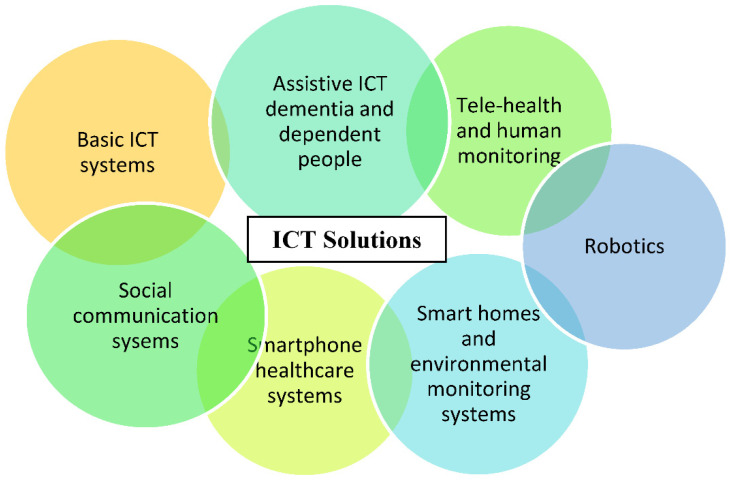
Different categories of ICT solutions from the simplest technologies (e.g., basic ICT) to the more complex solutions (e.g., smart homes and telehealth).

The range of ICT technologies varies between quite simple and very sophisticated ICT solutions, all having the common objective of improving the QoL of older people. Such ICT solutions can range from smart home systems and telehealth applications to reminder functions, fall detection/prediction/prevention systems, smartphones, etc. [35]. Figure 5 gives the different categories of ICT solutions.

Older people already use a wide range of ICT solutions in their daily lives. Such ICT solutions that are home-based, as televisions and microwave ovens, as well as computer-based technologies as internet and emails are considered Basic ICT Technologies. Basic ICT solutions have a positive impact on the QoL of older people living in their homes [36]. For example, internet-based technologies allow changes in older people’s health behaviour using different interventions as self-help programs and customised health-related data presentation through matching personal data to reachable interventions.

Social Communication Systems as mobile phones allow older people to stay in touch with their family members, caregivers, and clinicians or care providers. Social communication systems benefit older people living in their own environment by permitting social interaction using ICT solutions as video calling, thus decreasing isolation and depression levels in the older population as well as affecting older people’s health and level of life satisfaction positively [37]. Despite the advantage of allowing remote social communication, it is feared that ICTs could elevate the feeling of loneliness of older people living in their own environment [38]. Video conferencing ICT systems are also considered social communication systems. An example of such a system is those supporting older people with chronic diseases. These systems provide access to older people’s support around the clock and deliver a user-centred social care system interconnected to tele-medicine processes [39].

Smartphone Healthcare Systems give older people the opportunity to access their own health data and collaborate in their own healthcare process through accepting services that improve their health behaviour. These systems provide older people with lifestyle guidance, fitness exercises, chronic disease management, as well as providing public health surveillance [40].

Smart Home Solutions and Environmental Monitoring Systems provide older people with a collection of procedures based on various devices and sensors displayed in their living environment. Such ICT solutions enable monitoring and management of older people’s health using telehealth applications with the aim of improving their QoL and supporting their physical independence. Examples of smart home solutions and environmental monitoring systems can be fall detection systems, daily activity monitoring systems, and medical condition monitoring with vital data analysis. Such solutions address clinical syndromes through providing assurance and emergency assistance to the user and thus reducing caregivers’ burden [41]. These systems allow the older user to be more involved in decision-making rather than being only the recipient of clinical decisions taken by clinicians [42].

Robotics is expected to play a major role in the healthcare of older people in the future [43]. Robots vary in the role they perform in helping the user to live actively and independently in their own environment. Robots fluctuate between service robots that support older people to perform daily activities and robots that act as social companions. Robotics is mainly applied in systems addressing mental health conditions since it has an impact on emotional, physiological, and social health [44].

Telehealth and Human Monitoring are addressed mainly to home hospice people and their caregivers. They provide different monitoring services such as functional services as sleep quality monitoring, safety services as detecting environmental hazards, physiological services as monitoring vital health parameters, and security services as alert alarms. Such monitoring technologies aim to increase the independence of the user through allowing real-time intervention just in time and provide him/her with acceptable support. Telehealth and human monitoring solutions enable the empowerment of the older user, the caregivers, and family members by permitting their involvement in the daily care process [45].

Assistive ICT for Dementia and Dependent People provides older people with chronic diseases as dementia with more independence, safety, social communication, and an enhancement in activity performance. Thus, such solutions improve the QoL of older people living in their own environment. Older people using these systems are provided support in different areas of delivery of information. The provided support can be concerning their dementia symptoms, social interaction, health monitoring, and general safety. This type of ICT solution can increase its users’ self-confidence and reduce levels of social isolation. These systems are proven to increase the feeling of safeness and reduce the feeling of fear and anxiety in older people with dementia [46].

### 3.3. Development of ICT Solutions

**Figure 6:**
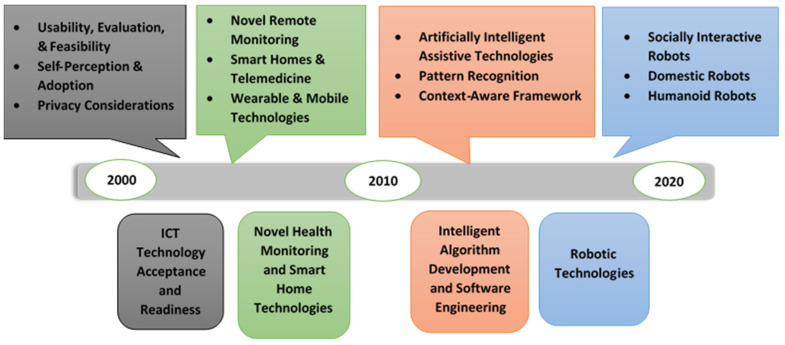
A detailed view of the development process of ICT solutions with its different aspects.

The main four themes that emerged throughout the development process of ICT solutions are: technology acceptance and readiness, novel patient monitoring and smart home technologies, intelligent algorithm and software engineering, and robotics technologies [47], as presented in Figure 6.

ICT Technology Acceptance and Readiness involves studies that took place in the early 2000s and is considered to be the first era of technology research for the aging society. In this phase, researchers examined older people’s acceptance of monitoring technologies using non-wearable sensors such as electronic health systems and smart home-based technologies using bed, motion, kitchen safety, and fall detection [31,32]. In addition to older people’s acceptability, other studies investigated the usefulness, feasibility, and privacy of assistive tele-monitoring systems [33,34].

Novel Health Monitoring and Smart Home Technology are more sophisticated ICT systems that emerged after the year 2000. This type of system uses both wearable and non-wearable sensors. Such systems rely on interaction between sensors, mobile devices, cloud servers, and supervised machine learning approaches, novel protocols over SMS to allow communication between the older user and caregivers [35,36]. It was recognised that the blending of telemonitoring intervention with smart home technologies enhanced older people’s skills in problem solving and self-efficiency in managing their medical conditions [37].

Intelligent Algorithm Development and Software Engineering became of great interest for research after 2010. This period is the second era of technology research for the aging society. In this phase, researchers explored the development of prototype systems, the establishment of new sensor-based smart home technologies, assistive robots’ development, and the conceptualisation of new AI and machine learning solutions. This development in technology led researchers to the capability of developing new sophisticated AI algorithms and advanced context acquisition methods for advanced analysation and automation of complex tasks [30]. An example of different intelligent algorithms is those involved in collecting data about the user’s environment and can predict potential problems for better decision-making (data mining algorithms). Examples of software development include the usage of context-aware middleware that can sense and respond to the user’s environment, the employment of pyroelectric sensors and infrared optoelectronic components with the aim of detecting electromagnetic radiation, and analysing the collected data to monitor and manage user’s daily activity [38,39,40].

Robotics Technologies emerged around 2010. There are various robotic technologies that affect older people’s healthcare. They vary between advanced AI robotic technologies and simple assistive robotic activities [40,41]. Telepresence robots, companion robots, home automation and domestic assistive robots, rehabilitation and health monitoring robots, and reminder robots are examples of robotic systems that can be integrated with older people’s healthcare monitoring and management solutions. It is revealed that the development and multidisciplinary nature of robotic systems played an important role in providing better interaction with the aging population as well as contributing to therapeutic benefits [30].

Over the last decade, different ICT solutions concentrating on AI and machine learning or focusing on the development of context-aware and adaptive technology rapidly emerged. The systems concentrating on AI and machine learning and using sensors and smart home devices have the aim of investigating user perception, barriers, and novel system development. Other systems focusing on the development of a context-aware technology have the aim of being integrated into any environment, collecting information from different sensors and devices concerning temperature, geographic location, and user preference as well as delivering relevant data based on the older user’s unique set of variables. Sapci et al. [47] announced that the use of AI and sophisticated algorithms in the healthcare monitoring and management of older people can improve the accuracy and progress of the analytical techniques performed, thus making the monitoring and management process faster and more accurate.

### 3.4. Examples of ICT Solutions

ICT solutions can help older people to manage their chronic conditions on a day-to-day basis. The use of ICT solutions can handle age-related physical and cognitive impairments aiming to prolong the functional capacity of older people, delay their institutionalisation, and increase their autonomy and participation in society.

Employment of ICT solutions for the early detection of chronic conditions enables older people’s self-management of personal health records and use of a management tool that integrates different aspects of an older person’s healthcare, including medical, social, and emotional issues. Older people with chronic conditions living in their own environment can be provided with adequate medical care and guaranteed health services through the use of efficient ICT networks [48].

Such ICT solutions involve continuous remote monitoring of older people for the management of different health conditions as hypertension and blood pressure and improve the prediction of new health conditions, as well as older person’s obedience.

ICT solutions permitting older person-centred care for diabetes mellitus involve blood glucose monitoring by telemonitoring systems, internet applications, and mobile devices. Such ICT solutions includes smart algorithms to control blood glucose levels as decision-making processors.

ICT solutions allowing older person-centred care for chronic heart diseases provide the potential for health ambulatory monitoring and enable remote coordinated care by healthcare providers. The scope of advance monitoring includes electronic weight scales, blood pressure (BP) meters, thermometers, as well as accelerometers for activity-monitoring and sleep-monitoring devices.

ICT solutions enabling older person-centred care for chronic obstructive pulmonary disease (COPD) include video or telephone links with healthcare providers either in real time or using store-and-forward technologies, internet-based telecommunication systems with healthcare providers, wired and wireless telemonitoring of physical parameters such as spirometry, respiratory rate, blood pressure, or oxygen saturation, pulmonary rehabilitation solutions with home-based video conferencing-supervised exercises and counselling, and telemonitoring of older people on home mechanical ventilation (HMV).

ICT solutions permitting older person-centred care for stroke mainly address physical care, consultation, and education. Such solutions provide monitoring services through integrating measurement devices, innovative interaction paradigms, customised motorial/cognitive neuro-rehabilitation treatments, continuous health status monitoring, cloud, and interoperable information systems.

ICT solutions allowing older person-centred care for cognitive impairment can support autonomous outdoor mobility, empowering participation in social events of older people with mild dementia. Such functionality is supported by assistive technology devices (ATDs), typically in the form of wearable devices which contain hardware and software that create a location-based service using global positioning system (GPS), cellular, and other signals. 

Fall prevention intervention ICT solutions include fall detection and prevention ICT services to help older people live independently in their own environment by providing assistive support to clinical decisions. These ICT solutions concentrate on reducing fall risks and help older people to overcome them. Hamm et al. [49] parcelled fall prevention ICT solutions into four categories.

Pre-fall prevention intervention ICT solutions are ICT solutions targeting older people at risk of falling but who have not experienced any falling event yet. Such ICT solutions focus on the functional ability of the older user, supporting older people at risk of falling with services such as targeted physical activities and educational programs. These solutions have the aim of overcoming various intrinsic fall risk factors as balance, muscle strength and cognitive decline, and vision [8,50,51]. In this category of ICT solutions, gaming consoles as Wii and Xbox are deployed. For this reason, this category of fall prevention ICT solutions was excluded from the review.

Post-fall prevention intervention ICT solutions are ICT solutions focusing on older people who have previously experienced a fall by providing support to eliminate their risk of encountering future falls [47]. This category of fall prevention ICT solutions includes diagnostic assessment functions aiming to deliver re-active intervention to its users. These solutions include fall prediction solutions in this review.

Fall injury prevention intervention ICT solutions are ICT solutions that aim to enable assistive communication between older people and their care providers or clinicians. These systems include fall detection systems in this review. Fall injury prevention intervention ICT solutions target three main intervention types: activity monitoring, fall detection, and medical assistance. These solutions include fall detection solutions in this review.

Cross-fall prevention intervention ICT solutions are ICT solutions that support older people by delivering a blend of the previously mentioned fall interventions. This combination of different interventions has the aim of helping older people to manage their health conditions and to live independently in their own environment, using different ICT services that permit assistive collaboration between all older people, caregivers, and care providers. This category of ICT solutions includes fall prevention solutions in this review.

## 4. State of the Art and Research Results: ICT Healthcare Solutions for Assistive Living in the Aging Population

### 4.1. Research Question and Its Impact on the Analysis Process

This review sought to analyse health-related ICT solutions for the aging society. The performed analysis was based on our research question of whether the available health-related ICT solutions are effective, reliable, and acceptable in terms of encouraging active and independent living in older people, satisfying their needs, improving their QoL, as well as making it affordable [9]. There are several prototype and commercial ICT solutions for pervasive healthcare monitoring for older people with chronic diseases. It is observed that the focus categories include cognitive decline and mental health, fall detection, prediction and prevention, heart conditions, chronic obstructive pulmonary disease, diabetes, and strokes. Table 1 lists ICT solutions covered in this systematic review under each medical condition. These technologies having the main aim of detecting changes in vital signs of the older person’s health do not need to be embedded in the living environment [41,52].

In a systematic review of the available ICT healthcare solutions for assistive living of older people between 2010 and 2014, ICT healthcare solutions were classified based on the major chronic illnesses of older people such as cognitive decline and mental health [55], monitoring heart conditions [73], obstructive pulmonary disease [80], fall prediction [91], and disease/disability prediction/health-related quality of life [109].

In another systematic review of the ICT healthcare solutions proposed between 1990 and 2018, the technologies were classified as either having the purpose of extending and sustaining older people’s independent living in their own environment [12,14,15,59,62,65,66,68,69,76,78] or assisting and simplifying caregivers’ activities [13,53,55,59,61,62,74,82]. It was stated that most of the discussed solutions were connected to specific chronic diseases as Alzheimer disease and other dementias, diabetes mellitus, and stroke. With very few exceptions, most of the available ICT solutions were either in the research and development phase or early prototype phase.

### 4.2. Results of the Analysis

Our review determined that the analysed studies focused on accepting available ICT technologies, developing new healthcare-monitoring and smart home technologies, enabling real-time channelling of data, and creating and integrating AI algorithms into ICT solutions. In this systematic review, the articles investigated included either randomised control trials (RCTs) or qualitative research of healthcare monitoring and management ICT solutions.

Table 2, Table 3, Table 4, Table 5 and Table 6 cover ICT solutions for assistive living of older people with different medical conditions: cardiovascular disease, cognitive impairment and other dementias, COPD, diabetes, and stroke. In each table, the ICT solutions belong to different time frames and use different categories of ICT technologies or multicomponent ICT interventions.

An in-depth analysis is carried out regarding the sensing technologies, smart technologies, and novel methods used in ambient assistive living (AAL) of older people with chronic diseases. Other aspects discussed in the analysis are the user interface, the older user, as well as the maturity level of the available ICT technologies. Finally, this paper reviews the older people’s acceptance and satisfaction of the discussed ICT technologies.

Table 2 provides examples of different categories of ICT solutions for older people with COPD. We selected a few articles discussing ICT solutions for older people with COPD as in Antoniades et al. [77]. The COMMONWELL project [76] allowed the combination of social care and healthcare for older people with COPD through developing an integrated e-care solution. Both client and server web services were implemented following a common interface. The services implemented provided an interface to exchange information such as username, ID, gender, social security number, call reason, user’s current medication, diseases, and allergies, etc. The proposed ICT solution providing telecare services for COPD patients showed positive effects on the older users’ mental health. The user’s feeling of security increased, whereas hospital admissions decreased throughout the study duration. The KERSA project [79], on the other hand, used sensors and devices, speech recognition and generation methods, robotic services, person-aware navigation techniques, an audio and video communication service, and eye contact and emotional monitoring to assist older people with COPD in living independently. It integrated smart home technology with assistive robotic services to serve older people’s needs.

Table 3 provides several ICT solutions for older people with heart conditions that use different approaches. The V2me project [15] developed an ICT solution for older people with heart conditions. V2me (Virtual coach reaches out to me) was an Ambient Assisted Living Joint Program (AAL JP) project that involved social network activities to enhance the quality of life of older people. The V2me project idea came up within another AAL JP project called A2E2 (Adaptive ambient empowerment of older people). The A2E2 project included a virtual-coach-assisted system that supports older people having diabetes type II and cardiovascular diseases with their lifestyle management. The developed system was implemented on an all-in-one PC having a touchscreen located in the older person’s home. The V2me system aimed to tackle aspects of the social behaviour of older people, mainly loneliness. It developed a user-centred designed system and implemented it on touchscreen devices to diminish loneliness in older people. Hervas et al. [72], on the other hand, developed an end-to-end software application that monitors the risk of heart diseases in older people, integrating a smartphone, sensors, high response speed, and rule-based decision support services in on system. This system benefits all the older persons, caregivers, and care providers. Kantoch et al. [75] developed a cardiovascular risk-based system that automatically recognises any inactive behaviour related to the heart and quantitatively measures older people’s physical activity. The system collected data from multimodal sensors, used a machine learning algorithm for activity recognition, and applied an experimental protocol for validation.

Table 4 consists of a group of selected ICT solutions that made a difference in the assistance of older people with cognitive impairment and other dementias as in existing works [44,56]. The ISISEMD project [110] provided a services package that allowed caregivers to remotely support older people whilst tolerating ethical rules and permitted family members to communicate and support older people with cognitive impairment and other dementias in their independent living. This system deployed environmental monitoring and control, a mobile localisation system, a tele-care service, reminder functions and brain games, and communication and videoconferencing. Alberdi et al. [64] developed a smart home-based system to detect multimodal symptoms related to Alzheimer’s disease. This system used sensors, devices, environmental sensors, and machine learning software to evaluate mood and cognition. The developed system measured the level of an older person’s mobility using the Arm Curl test. It detected changes in the older person’s cognitive and mobility skills. The model discussed is an early model. The need for collecting more data and integrating machine learning algorithms is intended as future work with the purpose of building accurate prediction models and adjusting to imbalanced detection problems prior to the system’s implementation in the real world.

Table 5 provides selected ICT solutions for older people with diabetes mellitus as in existing works [81]. Earle et al. [84] developed a mobile telemonitoring system for older people with diabetes; this system has the potential for delivering intensified care to improve blood pressure control, and its use may be associated with reduced exposure to hyperglycaemia. Blood pressure readings were transmitted wirelessly via sensors in the mobile phone to the central server. This system allowed real-time transmission of data between users and care providers using a web-based application delivering management services. Salvo et al. [84] developed a smart wearable and autonomous negative pressure device that benefits from temperature sensors and pH sensors to monitor and manage chronic ulcers over time. In laboratory settings, the developed system proved to be suitable for pH measurement but still needs clinical validation.

Table 6 provides selected ICT approaches for the assistance of older people encountering strokes. Pastorino et al. [67] developed a CogWatch framework to evaluate a platform for the personal rehabilitation of older people that encountered a stroke. This framework consisted of the monitoring device, CogWatch, the Client Sub-system (CCS), and the CogWatch Professional Interface (CPI). The developed system was designed to monitor user’s movement and task execution data and recognise the presence of any action error. This monitoring system used a set of sensors, cameras, a kinetic device, servers, web application, and a user interface. Oliver et al. [70] developed an ambient intelligence environment for the home cognitive telerehabilitation of older people. This system aimed to provide users with more self-reliance, using a headset for emotional detection, a Kinect version 2, a Glove vibrotactile sensor, servers, and web-based services with client applications.

Most of the ICT solutions used for the different chronic diseases use web or mobile applications that need to be more interactive and user friendly. Several solutions consider speech recognition to process user input. A large group of the solutions considered non-invasive sensors (in terms of privacy and personal space) which are creditable. Almost all the solutions consider the older person as the user and consider a collaboration between the older person, caregiver, and, in some cases, the care providers. It is noticeable that almost all the ICT solutions discussed are at the R&D or prototype level. Most of the solutions did not report technology acceptance or user satisfaction. The solutions ranged between health monitoring and management and activity monitoring with the inclusion of an alert system or reminder service for users.

Table 7 provides fall prediction ICT solutions for assistive living of older people. Fall prediction interventions [93,94,99,100,101] delivered functional, cognitive, and environmental assessment to the user. Majumder et al. [96] developed a smart phone-based fall assessment system by integrating sensor data from a smartphone and a smart shoe. The developed system collected the user’s data in any environment using a smart shoe containing four pressure sensors with a Wi-Fi communication module. This system monitored abnormal gait patterns while performing ADLs. Staranowicz et al. [97] proposed a mobile Kinect-based gait-monitoring system for fall prediction that used an autonomous robot which monitored the walking patterns of older people during their ADLs at home and recognised functional decline.

Most of the proposed fall prediction systems did not provide the user with an interactive interface, which implies their static nature. Majumder et al. [96] and Almer et al. [98] developed static applications that used smartphone sensors to collect fall relative data from older people’s behaviour while living in their own environment.

Table 8 presented fall detection ICT solutions for assistive living of older people. Fall detection systems included solutions [25,87,88,89,90] that focused on eliminating after fall injuries. Abbate et al. [88] proposed a fall detection system that integrated an artificial neural network and feature extraction machine learning methods to monitor the older person’s movement behaviour and produce emergency alerts after a fall was detected. Wang et al. [92] proposed a refined fall detection system that used on-body smart sensors operating in the older person’s living environment to monitor their movement behaviour. This system focused on the common changes in the user’s movement behaviour accompanying an accidental fall. These include changes in impact magnitude, trunk angle, and after-event heart rate. It collected and analysed data from an accelerometer, smart sensors, and a cardiotachometer to accurately distinguish between older people’s ADLs and fall-related behaviours.

Most of the presented fall detection solutions developed data filtering methods to distinguish fall-related behaviour from ADLs. These systems function by gathering older person’s movement data profiles, detect a fall and send automated alerts to caregivers and/or care providers, and filter false detection.

There appear to be no fall detection solutions deployed on game console platforms. Both Abbate et al. [88] and Abbate et al. [89] developed on-body fall detection solutions. These solutions were implemented on a smartphone that needed to be worn by the older user as a wearable device. Most of the presented fall detection ICT solutions were set up from three fundamental components, i.e., included a device with embedded wearable sensors to collect physiological data from the older user, a filtering method to distinguish fall related events from ADLs, and a form of communication in case of emergency [111].

To detect a fall, a range of information sources need to be exploited. The location of the sensor on the older user or in the living environment is important for the fall detection process. Terroso et al. [90] developed a fall detection system that collected users’ movement data using a wearable accelerometer and sent it to the smartphone and server for further analysis. The accelerometer sensor communicated to a smartphone application through Bluetooth to perform the analysis. The geographical location of the user was logged using a smartphone GPS sensor. The system delivered messages to the caregivers in case of emergencies. In other works (for example, Kepski et al. [86]), context was the main information source used. Data were conservatively collected from older people and this is considered less intrusive than approaches with sensors placed on the user’s body. This approach used a Kinect to detect falls of older people in their living environment. This system performed 3D tracking of the users but had some limitations as limited spatial coverage and inability to monitor user’s movement while walking.

Table 9 provides cross-intervention fall prevention ICT projects for assistive living of older people with chronic diseases. Shi et al. [102] developed a fall prevention system for the assessment of fall risks using a smartphone application. This system detected falls after their occurrence using clinical tests for the purpose of preventing fall-related injuries. Chou et al. [103] presented another type of fall prevention system. This system detected changes in the position of the user from lying to sitting while getting out of bed and then sent alarm signals when a risk of falling was detected, allowing immediate support by caregivers.

The previously mentioned cross-fall prevention intervention systems utilise a full range of techniques that correlate with both fall prediction and detection with the target of assessing, detecting, and responding to fall risks. In this systematic review, we do believe that the wider the range of weaknesses, risks, disabilities, and diseases monitored in an older person, the more results we obtain out of the cross-fall prevention intervention. Different qualitative fall prevention approaches were developed throughout Europe. I-DON’T_FALL [106] is a project that analysed a fall prevention system for older people with cognitive impairment. This system showed positive effects on older people’s quality of life through reducing their fear of falling and the risk of falling and enhanced the older people’s mobility. ISTOP-FALLS [107] is another project that focused on fall detection and fall-related factors that promised to provide a fall prevention system with a wide range of services including a fall risk assessment and prediction system and a personal health advisor. The large international AAL project ReAAL [108] encompassed older people residing in newly developed assisted living homes equipped with AAL systems. The developed solution developed an emergency calling service, a social communication service, and an environmental control service through developing 32 applications in different pilot sites with the aim of including a very large number of older people from different countries in Europe. These applications collected data from users as minimal datasets, surveys, real-time tests, issue reporting by user, etc. This solution was not tested by older people and faced many challenges on an organisational level, deployment level, as well as user recruitment and consent level.

## 5. Discussion

In this systematic review, 63 articles were selected according to their relevance to the research question and ICT solutions focusing on the biomedical perspective of aging. Then, an analysis of the objectives, impacts, and role of the presented ICT solutions in monitoring and managing the chronic diseases, ADLs, and falls and near-falls was performed. The analysis performed has sought for answers of whether the presented solutions were effective, reliable, an acceptable by the aging population. Most of the selected papers discuss ICT solutions connected to cognitive impairment and other dementias (16×) and fall interventions (24×), while other chronic diseases were represented by 5 to 7 articles each (Table 1).

In recent years, there has been a high demand for monitoring older people with any kind of dementia [11,55,59]. AAL technologies [57] as well as smart home systems [55,66] have been extensively proposed as effective ICT solutions for older people with dementia and their caregivers. With the objective of a more independent life, these solutions monitor the older people using passive and active IR motion sensors [59] or video monitoring systems [11,79] depending on the level of accuracy required.

Diverse presented research papers discussed and evaluated the use of ICT solutions for AAL of older people. A group of papers discussed the older people’s ability and acceptance of using tablets and touchscreen devices [15,60,62,68]. Hwang et al. [60] explained that the developed ICT solutions need to be flexible, customisable, and have the feature of Do It Yourself (DIY) to enhance the older people’s healthcare, experiences, and relationships while living in their own environment. Muuraiskangas et al. [15] emphasised the importance and the necessity of the user’s involvement throughout the development, implementation, and testing phases of the developed ICT solutions to enable a smooth transfer of the ICT solutions from virtual settings to the real world.

Currently, the use of multicomponent interventions and smartphones is becoming a trend. It is reported that older people living in their own environment have the ability of using different ICT solutions as smartphones and wearable devices [81]. Web or mobile applications are generally used in most of the discussed ICT solutions for speech recognition. It is observed that non-invasive sensors and smart capabilities are becoming a trend in new ICT solutions.

Almost all ICT solutions consider the older person as the user. In general, the older person, caregivers, and/or healthcare providers are intended to use the system in collaboration. The state of development of ICT solutions in most of the publications is either R&D or prototype phase. This incompleteness in the development process of the solution is predictable considering the nature of the problem, where almost all discussed solutions did not announce older people’s acceptance and satisfaction in the developed ICT solutions. This problem is caused due to the older person’s distrust in using the developed ICT solutions in their daily life, as mentioned in many research papers [17,67,72,90].

Almost all ICT solutions as the ones connected to fall prevention focused on deploying machine learning techniques and advancing AI algorithms to improve fall detection solutions’ levels of accuracy, specificity, and sensitivity. Very limited effort was applied on the design and user interface of the developed ICT solutions. Most of the deployed applications were static, having the main role of detecting falls as they occur. The presence of interactive applications to interact with the users to reduce fall risks was not observed in most developed ICT solutions.

It is important to mention that the older people need to be willing to wear the device in the first place. The user may consider solutions with built-in accelerometers and gyroscopes, as the one developed by Abbate et al. [89], but they are intrusive and expensive. Alternatively, there are other presented solutions that used camera sensors [86], pressure sensors, and audio sensors or brand-specific sensors as Microsoft Kinect. Kepski et al. [86] developed a fall detection system that reused a Kinect to collect older people’s data while living in their own environment.

Almost all the presented ICT solutions used multimodal interaction to collect data from the user, control the system, and allow interaction between the users, caregivers, and/or care providers using interaction devices. Most approaches used non-interactive interfaces, while only some systems presented [88,89] used both non-interactive interfaces and touchscreen gestures.

As for fall-related ICT solutions, the fall detection and prediction literature appears to be overloaded with solutions that monitor user’s activity, detected falls, and sent alerts. Nevertheless, there are some challenges associated with these solutions and their use in practice. There is a need for more accurate detection of falls with an advanced ability to distinguish fall events from ADLs. Another challenge is preserving the user’s privacy. One way of preserving the user’s privacy is the use of camera sensors instead of wearable sensors.

In almost all existing ICT solutions, artificial intelligence employment was reported. This means that any increase in the quantity of the user’s collected data will have a positive impact on the developed ICT solution.

The smart capabilities, the non-intrusiveness, and the possibility of interaction between older people, caregivers, and/or healthcare providers, in the discussed ICT solutions, highlight the potential that these solutions have in enhancing the quality of life of older people with chronic diseases and maintaining their health and wellbeing [17].

Finally, it should be noted that the findings of this review cannot be generalised to the heterogenous aging population. Thus, depending on the heterogeneity of an aging population, different approaches and technologies are required based on the diverse needs of the specified aging population. This indicates a key limitation facing the wide adoption of technology in heterogeneous aging populations. Our foundation in addressing this limitation would be the development of smart gateways or interfaces to be adaptive, learn from the user, and address each user’s unique biomedical and chronic needs.

## 6. Conclusions

Based on the literature review performed, the currently available ICT solutions considered the subtleties of the disease instead of the wider context, i.e., the chronic disease in correlation with the older person’s unique health and capabilities profile and the unique characteristics of the lived-in environment. Frequently, many of the available ICT solutions are used along the chronic diseases and disabilities rather than managing the healthcare and abilities profile of the older person as a whole [17].

After analysing the various healthcare monitoring and management ICT solutions, different research gaps were identified and stated here. The existing ICT solutions do not tackle the fusion process of data captured using wearables from both contextual and physiological sensors. New data sources need to be identified and more comprehensiveness in the developed solutions is required. This implies the need for future research on more efficient and sophisticated means of collecting, managing, and analysing data and delivering medical information to caregivers and care providers, helping to drive data-informed health care decisions. More insight into the use of data analytics and health informatics tools as machine learning, database management, cloud computing, predictive analytics, and data visualisation is required in future research. The developed ICT solutions are in need of user-friendly interfaces and feedback techniques for more engagement and empowerment of the older people in their own healthcare management process. There is a need to address user interface effectiveness, efficiency, and satisfaction by older people in future research. Although healthcare providers or clinician’s interactions were included in some of the developed ICT solutions, there is a need for interactive, comprehensive, and powerful web interfaces to permit effective visualisation of older people’s health data and provide helpful support [112].

Therefore, deploying comprehensive, interactive, and effective ICT in areas of assisted healthcare enables older people living in their own environment to perform unsupervised contributions with remote monitoring by healthcare providers, but still there is no evidence of the effect of ICT solutions on older people’s health outcomes.

Identifying the challenges facing the current healthcare monitoring and management solutions for older people makes the direction of future research more evident. In this regard, the presence and the involvement of ICT solutions in assisting older people with daily activities in their daily life is increasing. Nevertheless, older people are unwilling to utilise ICT solutions with wearable sensors due to privacy and security concerns [66]. The exhaustion of using wearable technologies is another reason for the unacceptability of these technologies by older people. There is thus a need for a balance in means of accessing and/or sharing the users’ data while maintaining their privacy and security by controlling older person’s data accessibility, recognition of potential attacks by security and privacy providers, and ensuring that wireless networks and messaging systems are secured. The developed ICT solutions need to be more personalised to the user’s requirements, more pervasive, and multifunctional [113,114].

This leads to the identification of user-oriented requirements of smart ICT solutions. They need to be designed and implemented targeting user’s acceptance and satisfaction of the developed solutions. To fulfil the need of helping older people manage their chronic diseases and disabilities while living in their own environment, a set of requirements must be addressed by the ICT solutions [115]. Older people are not comfortable with the technology being provided due to its unusefulness to them or due to the increase in their feeling of being stigmatised and disabled. ICT solutions should not be obstructive, thus leading to disuse by the user. The user’s privacy and security while using the solution need to be boldly recognised and understood by the user. The ICT solution must be affordable to be owned by older people. Finally, and most importantly, the developed ICT solutions need to support older people in staying in their own environment as well as moving in various environments.

Based on the reviewed literature, one can conclude that the topics of disability prediction, health-related and independent QoL, and fall prevention are not adequately covered in the developed ICT solutions. It is concluded that the developed ICT solutions are not flexible, adaptive, or user-oriented. They do not provide assistive support for any health or disability predictions, and none of the developed ICT solutions can be considered to be low-cost solutions [17,109,112].

## Figures and Tables

**Table 1:** Conditions/behaviours addressed by technologies (n = 63).

Medical Condition and Disability Addressed	Number of Papers	Study
Cognitive decline and mental health	16	[11,12,13,47,53,54,55,56,57,58,59,60,61,62,63,64]
Stroke	6	[65,66,67,68,69,70]
Monitoring heart conditions	6	[15,71,72,73,74,75]
Chronic obstructive pulmonary disease	5	[76,77,78,79,80]
Diabetes mellitus	6	[14,81,82,83,84,85]
Fall detection	7	[26,86,87,88,89,90,91,92]
Fall prediction	9	[93,94,95,96,97,98,99,100,101]
Fall prevention	7	[102,103,104,105,106,107,108]
**Total**	**63**	

**Table 2:** ICT solutions for assistive living of older people with COPD.

Study	Project Name	Year	Type & Design	Objective	Findings	Limitations
Antoniades et al. [77]	COMMONWELL Project: Early Intervention and Telehealth for COPD Patients—Milton Keynes.	2011	Pilot study. Telecare equipment (social alarm) and telehealth monitors in patients’ homes. Telehealth readings and alarm calls at a joint call centre.	To improve the care for older people with COPD through an integrated telecare and telehealth service.	Developed and implemented integrated services supporting effective management of chronic diseases and addressed reduced mobility, vision, or hearing.	Not mentioned
Pedone et al. [78]	“SweetAge” monitoring system: Efficacy of multiparametric telemonitoring on respiratory outcomes in older people with COPD.	2013	RCT: A randomised controlled trial. Placed on the user’s body. Consists of a wristband with sensors, a pulse-oximeter, a mobile phone.	Telemonitor user’s oxygen saturation, heart rate, near-body temperature, and physical activity to evaluate the solution’s efficiency in reducing hospitalisation of older people with COPD.	The study showed the ability to detect respiratory events quickly using an automated wearable system that is easy to use by older people. It is concluded that the solution eliminated the risk of hospitalisation by 33%.	Lower incidence of events, large confidence interval around the focus point.
Johnson et al. [79]	KERSA Project	2013	KERSA solution included a robot controller to control robot behaviour, a rule engine, and three databases. The system ensures connection and coordination between the three components.	Provide a framework to enable independent living of older people in smart homes through functional and social interaction with a robot.	The study fulfilled the older people’s needs through providing the following services: health and behaviour monitoring using a mobile robot, environment monitoring using a robot integrated with smart household technology, provide audio and video communication services with caregivers.	Robot was expensive and lacks the ability to navigate in domestic environments.

**Table 3:** ICT solutions for assistive living of older people with heart conditions.

Study	Project Name	Year	Type & Design	Objective	Findings	Limitations
Wade et al. [71]	Telemonitoring With Case Management for Seniors with Heart Failure.	2011	RCT. A randomised clinical trial. Designed to allocate older people with high-risk using case management (THCM) versus case management (CM) alone.	Allow health management of older people with risk of heart failure by providing nurse case management services through telemonitoring.	Effective implementation of heart failure telemonitoring intervention.	The developed solution had no impact on lower levels of overall morbidity and mortality.
Muuraisking et al. [15]	V2me Project Evaluating the first steps in mobile friendship coaching.	2012	Pilot evaluation report—A prototype of V2me system.	Develop a user-centred design addressing loneliness issues in older people through the use of an easy-to-use technology including touchscreen devices.	The importance of constant older person involvement throughout development, implementation, training, and support while using the developed system.	The final product was useful only to older people that have pronounced needs.
Hervas et al. [72]	Mobile monitoring and reasoning methods to prevent cardiovascular diseases.	2013	Use of mobile phones and sensors with Bluetooth communication. An integrated rule-based decision support solution.	To monitor risks of heart diseases in older people using mobile phones through developing an end-to-end software application.	Developed and evaluated an end-to-end solution for monitoring that can be used by patients, caregivers, and doctors and uses smartphones.	The developed solution showed only 69% acceptance rate by older people.
Rifkin et al. [73]	A randomised, controlled clinical effectiveness trial of real-time, wireless blood pressure monitoring for older patients with kidney disease and hypertension.	2013	RCT. Telemonitoring device that pairs Bluetooth-enabled blood pressure cuff with an internet-based hub.	To monitor blood pressure of older people at home to improve blood pressure management of older people with kidney disease.	Greater sharing of data between older people at home and their clinicians using a low-cost wireless monitoring method. Improvement in blood pressure management.	The study had small sample size and was performed over a short duration.
Kantoch et al. [75]	Recognition of Sedentary Behaviour by Machine Learning Analysis of Wearable Sensors	2018	A prototype of a designed shirt for activity recognition. Development of a method for gathering data from multimodal sensors and machine learning algorithms.	Obtain qualitative measurement older people’s physical activity throughout developing a method for sedentary behaviour recognition.	High accuracy in detection of sedentary behaviour using smart shirts and machine learning techniques.	The detected set of activities is limited, advanced research is required.

**Table 4:** ICT solutions for assistive living of older people with cognitive impairment and other dementias.

Study	Project Name	Year	Type & Design	Objective	Findings	Limitations
Mitseva et al. [110]	ISISEMD Project Intelligent System for Independent Living and Self-care of Seniors with Mild Cognitive Impairment or Mild Dementia.	2009	RCT: One-year randomised control trial in real-life conditions in four European countries. Pilot evaluation report.	Provide support for older people enabling them to live safely at home, supporting them with daily basic activities, permitting their social interaction with family members and caregivers to prevent their social isolation.	Delivered environmental monitoring and management services. Provided a mobile localisation system. Assisted older people with reminder functions and communication and videoconferencing services.	Solution was in initial phases. System was not yet installed in the selected pilot sites.
Gellis et al. [58]	ALFRED Project Personal Interactive Assistant for Independent Living and Active Ageing.	2013	The ALFRED system consisted of three major sub-systems: - Client, on mobile device. - Server components. - A web portal.	- To empower older people to live independently. - To prevent age-related physical and cognitive impairments. - To improve caring by offering direct access to vital signs for caregivers.	Provided older people with user-driven interaction assessment, personalised social inclusion, personalised and effective care, and prevention of physical and cognitive impairment.	Not mentioned
Hwang et al. [60]	Co-Designing Ambient Assisted Living (AAL) Environments: Unravelling the Situated Context of Informal Dementia Care.	2015	A user-centred design (UCD) including a model that studied users, designed a problem space, and built and evaluated prototypes.	- To identify and address the activities and situations for which older people needed AAL support. - To describe how older people can specify and obtain their needs.	Developed an activity-assistance AAL solution providing video demonstrations for older people and personalised the developed AAL system to older people’s unique needs.	Sample size was small, included only females from one community-based support agency.
Alberdi et al. [64]	Smart home-based prediction of multi-domain symptoms related to Alzheimer’s Disease.	2018	A regression analysis of smart home-based behaviour data. Longitudinal smart home data labelled with corresponding activity classes and extracted time series statistics containing 10 behavioural features.	- To develop tools for detecting earliest stages of age-related disorders such as Alzheimer’s Disease. - To evaluate the possibility of using unobtrusively collected activity-aware smart home behaviour data to detect multimodal symptoms to be impaired in AD.	- Created regression models to predict symptoms. -Built classification models to detect reliable absolute changes in the scores. - Used SmoteBOOST and wRACOG algorithms to overcome class imbalance. - Showed that activity-aware smart home data can predict mobility, cognition, and depression symptoms in the older person.	Early models of the system. Results require completion and improvement.

**Table 5:** ICT solutions for assistive living of older people with diabetes mellitus.

Study	Project Name	Year	Type & Design	Objective	Findings	Limitations
Costa et al. [83]	VirtualECare: Group Support in Collaborative Networks Organizations for Digital Homecare	2009	A study on the introduction of collaborative networks in the care of the older people as Group Decision Support Systems (GDSS).	To monitor and allow interaction between older people and caregivers through developing an intelligent multiagent system interconnecting to other computing systems running in different healthcare institutions, leisure centres, training facilities, or shops.	Project in planning phase. Allow clinicians to achieve better analysis results from an older person’s Electronically Clinical Profile (ECP) by the development of Group Decision Support Systems (GDSS) in the healthcare.	Incomplete system. Multiple unrecognised data sources as unreachable sensors, incomplete user’s Electronic Clinical Profile.
Jara et al. [14]	Movital: An internet of things-based personal device for diabetes therapy management in ambient assisted living (AAL)	2011	Movital includes the module SkyeModule M2 from SkyeTek for contactless identification to identify patients, and load patient health profile, and the module Jennic JN5139 for Wireless Sensor Networks (WSN) communications.	It provided a diabetic older person with an electronic personal device that stores blood glucose readings and gave advice about the next meal and insulin injection.	Supported an older people’s profile management service implementing personal RFID cards, provided global connectivity between older person’s personal device and care providers or clinicians, and developed a desktop application to manage user’s personal health cards, glycaemic index information system, and user’s web portal.	Not mentioned
Salvo et al. [85]	SWAN-iCare (smart wearable and autonomous negative pressure device for wound monitoring and therapy).	2017	A study on monitoring diabetic foot and venous leg ulcers using temperature and pH sensors	To allow continuous monitoring of foot ulcers associated with diabetes.	pH sensor shown to be suitable for pH measurement in laboratory trials.	Sensors required clinical validation.

**Table 6:** ICT solutions for assistive living of older adults that encountered a stroke.

Study	Project Name	Year	Type & Design	Objective	Findings	Limitations
Pastorino et al. [67]	CogWatch project: Preliminary Evaluation of Personal Healthcare System Prototype for Cognitive eRehabilitation in a living Assistance Domain.	2014	Prototype phase. Designed to promote independence in activities of daily living for older people with apraxia. Included a Kinect and a set of sensors.	To collect data for executed tasks and movements of an older person to permit recognition of any action errors.	Showed 99% success in storing data correctly in terms of video records, tracking records, and streaming records. The ability to analyse the behaviour of external devices. Analysis of the behaviour of the external devices, i.e., Kinect, smart watch, and sensored tools.	System should be more flexible for use by older people, who required an improvement in the user interface.
Oliver et al. [70]	Ambient Intelligence Environment for Home Cognitive Telerehabilitation.	2018	A study that uses smart sensors, actuators, and a headset to implement a fuzzy inference system with an attempt to take place of a clinician.	To analyse the movement of upper extremities of an older person by capturing his/her body shape using a Kinect depth camera.	Provided both older people and their care providers with greater power and control.	Sample size was limited. Faced older people’s fear from the increasing dependency of rehabilitation on machine automation.

**Table 7:** ICT solutions for fall prediction systems for assistive living of older people.

Study	Project Name	Year	Type & Design	Objective	Findings	Limitations
Ferreira et al. [95]	A Pilot Study Testing a Fall Prevention Intervention of Older Adults.	2012	A descriptive study on monitoring healthy older people wearing sensors while performing different movements. System included five wireless placed on the user’s wrists, ankles, and chest.	To continually monitor and display older people’s movement and send real-time alarms for fall prevention. To use a standardised script to direct an older person’s movement with attached on-body sensors.	The system achieved high accuracy levels where sensor data output always matched video output.	Small sample size. Lacked accuracy and acceptance of hospitalised older people.
Majumder et al. [96]	Smart Prediction: A Real-time Smartphone-based Fall Risk Prediction and Prevention System.	2013	A study that integrated sensor data of smartphones and a smart shoe to predict falls. Smart shoe is designed to include four pressure sensors with Wi-Fi connection to collect data in any environment.	To recognise walking patterns and generate an alert message to the user in case of encountering high risk gait patterns to save them from near-falls.	Showed 97.2% accuracy in real-time detection of gait abnormalities in users.	Need to be tested on real older people with chronic gait problems.
Staranowicz et al. [97]	A Mobile Kinect-based Gait-Monitoring System for Fall Prediction.	2013	RCT. A randomised control trial to monitor human gait during daily-life activities through deploying a high-accuracy motion-capture system. It consists of Kinect and a robotic platform.	To allow collection of fall-prediction gait parameters without the use of on-body sensors with unlimited capture volume.	The system included a depth camera mounted on a robot that enabled high accuracy to be revealed.	Gait parameters and analysis need to be expanded. A comparison between fall predictions is required from clinicians.

**Table 8:** ICT solutions for fall detection systems for assistive living of older people.

Study	Project Name	Year	Type & Design	Objective	Findings	Limitations
Abbate et al. [88]	A Smartphone-based fall detection system	2012	A prototype of the system was tested on a small sample group. Used a small external sensing unit to eliminate the intrusiveness of the system.	Monitor older people’s movement patterns, detect a fall, and send automatic alerts for caregivers to provide support.	Proved that the recognition of fall-like activities of daily living (ADL) can significantly reduce the number of false alarms depending on peculiar features of the acceleration patterns of the user.	The small size of the sample group limited drawing conclusions with high confidence.
Abbate et al. [89]	MIMS: A Minimally Invasive Monitoring Sensor Platform	2012	A platform for the development of a health monitoring system based on older people’s needs. It included a virtual hub and a gateway. A gateway for communication of captured data enables coordination of signal processing and fusion of sensor data.	To predict emergency events through analysing the physiological signal data of the user. To allow communication of collected data, signal processing, and fusion of sensor data through using a gateway.	Optimised the quality of sensor data using a monitoring application that joined data acquisition and processing.	Long installation time was required, coverage capability was limited, and included privacy violations.
Terroso et al. [90]	A Wearable System for Fall Detection	2013	A study on the use of a system built up from a wearable sensor, a smartphone, and a website.	Provide higher autonomy to the older population, allowing for a more active lifestyle.	Sent an alert using the smartphone to family members or when a fall was detected. In case of unconsciousness, the alert message was sent after a pre-defined time interval.	Carrying the smartphone and having access to mobile network.
Cabestany et al. [91]	FATE Project	2013	Pilots are organised in three different countries under one year, involving 175 users. Includes accelerometers and gyroscopes for reliable fall detection.	To organise a big pilot on automatic fall detection of older people living at home.	An accurate, real-time detection of falls in older people.	Not mentioned

**Table 9:** ICT solutions for cross-fall prevention systems for assistive living of older people.

Study	Project Name	Year	Type & Design	Objective	Findings	Limitations
Siegel et al. [105]	ModulAAR	2014	Study protocol. A pre- and post-assessment, longitudinal, and quasi-experimental cohort study. Study duration was 18 months and included 2 pre-assessments in 7 different sites in Austria.	Use of ICT to help older people enhance their QoL through health improvement and independent living if possible.	Provided environmental monitoring and control, individual and health monitoring, a fall detection system with localisation and emergency services, reminder functions, social interaction application, communication, and video conferencing.	Not mentioned
I DON’T FALL [106]	I DON’T FALL Project	2015	A medical evaluation report and framework. A randomised control trial consisting of four arms. It detects falls and prevent injuries and was tested by over 500 older patients across Europe for 9 months.	Enable care providers to monitor older people more efficiently and cost-effectively and equip potential fallers with appropriate devices.	Key innovations: a robotic rollator—the i-walker. Delivered a fall detection system providing the older people with fall prevention services as warnings, and technical assistance for physical training through tele-rehabilitation.	Not mentioned
ReAAL [107]	I STOP FALLS Project	2015	RCT, 153 community-dwelling participants performed the program for 16 weeks.	Reduce older people’s risk of falling.	The developed program reduced user’s physiological fall risks. It showed an improvement in postural sway, stepping reaction, and executive function.	Small improvement in older people’s QoL was detected.
Liu et al. [109]	universAAL Project (make it ReAAL)	2016	An evaluation report of a qualitative longitudinal study involving pre- and post-interviews with older people moving to new assisted living homes.	The development of a single communication platform for digital purposes targeting psychologically less independent older people.	Provided an emergency call system, an environmental control system, and a social communication system.	Difficulties and delays in setting up application-level service provision.

## Data Availability

Data is contained within the article.
